# Silybin Showed Higher Cytotoxic, Antiproliferative, and Anti-Inflammatory Activities in the CaCo Cancer Cell Line while Retaining Viability and Proliferation in Normal Intestinal IPEC-1 Cells

**DOI:** 10.3390/life13020492

**Published:** 2023-02-10

**Authors:** Dominika Faixová, Marek Ratvaj, Ivana Cingeľová Maruščáková, Gabriela Hrčková, Viera Karaffová, Zita Faixová, Dagmar Mudroňová

**Affiliations:** 1 Department of Pharmaceutical Technology, Pharmacognosy and Botany, University of Veterinary Medicine and Pharmacy, 041 81 Košice, Slovakia; 2 Department of Microbiology and Immunology, University of Veterinary Medicine and Pharmacy, 041 81 Košice, Slovakia; 3 Institute of Parasitology, Slovak Academy of Sciences, 040 01 Košice, Slovakia; 4 Department of Morphological Disciplines, University of Veterinary Medicine and Pharmacy, 041 81 Košice, Slovakia; 5 Department of Biology and Physiology, University of Veterinary Medicine and Pharmacy, 041 81 Košice, Slovakia

**Keywords:** *Silybum marianum*, colon cancer, cell cycle, cytokine

## Abstract

The anticancer potential of silymarin is well known, including its anti-inflammatory as well as antiproliferative effect mediated by influencing the cell cycle, suppression of apoptosis, and inhibition of cell-survival kinases. However, less is known about silybin, the main component of the silymarin complex, where studies indicate its dual effect on the proliferation and immune response of various cell types in a dose-dependent manner. Moreover, there is a lack of studies comparing the effect of silybin on the same type of healthy and tumor cells, especially intestinal ones. Therefore, our study aimed to investigate the concentration-dependent effect of silybin on the normal intestinal porcine epithelial cell line-1 (IPEC-1) and the human epithelial colorectal adenocarcinoma cell line (CaCo-2). The metabolic viability, cell cycle, mitochondrial membrane potential, apoptosis, and the relative gene expression for pro- and anti-inflammatory cytokines were monitored in cells treated with silybin. Silybin stimulates metabolic viability as well as proliferation in IPEC-1 cells, protects the mitochondrial membrane, and thus exerts a cytoprotective effect, and has only a minimal effect on the gene expression of pro-inflammatory cytokines but significantly increases the expression of anti-inflammatory TGF-β. In contrast, it inhibits metabolic viability in tumor intestinal CaCo-2 cells, has an antiproliferative effect accompanied by increased apoptosis, and significantly reduces the expression of genes for pro-inflammatory interleukins as well as TGF-β. The antiproliferative and anti-inflammatory effect of silybin on tumor intestinal cells without a negative effect on healthy cells is a prerequisite for its potential use in the adjuvant therapy of colon cancer; however, further studies are necessary.

## 1. Introduction

Milk thistle (*Silybum marianum* L. Gaernt.) is a traditional medicinal plant. The first recorded mention of this plant is in the Old Testament (Genesis 3:18). It was used in clinical practice for liver dysfunction in Ancient Greece and for liver and gallbladder problems in Indian and Chinese medicine [1]. In the 1970s, silymarin (an extract of milk thistle fruits) was classified by the WHO as an official medicine with hepatoprotective effects [2].

The main potential compound of *Silybum marianum* is a silymarin complex, which contains mainly flavonoids (silybin, silychristin, silydianin, isosilybin, taxifolin, quercetin, dihydrokaempferol, kaempferol, apigenin, naringin, eriodyctiol, and chrysoeriol) and other compounds in very low concentrations (e.g., 5,7-dihydroxy chromone, dehydroconiferyl alcohol, linoleic acid, oleic acid, palmitic acid, tocopherol, cholesterol, campesterol, stigmasterol, and sitosterol). Silybin is the main component of the silymarin complex and is composed of two stereoisomers, silybin A and silybin B [3].

Silymarin complexes are well known for their proven therapeutic effects. In addition to its antioxidant and hepatoprotective effects, silymarin shows neuro-, nephro- and cardio-protective effects in animal and human trials. The new therapeutic potential of silymarin relates to its effect on degenerative brain disorders [4,5].

Moreover, silymarin has an immunomodulatory effect, which includes immunostimulatory as well as immunosuppressive activities. Johnson et al. [6] studied the impact of silymarin on differentiation and cell selection in the thymus via gene expression in Balb/c mice and confirmed that in vivo exposure to silymarin affects phenotypic selection processes in the thymus in a dose-dependent manner. The effect of silymarin on the immune system was confirmed to be concentration-dependent, as it suppressed T cell functions at low doses and stimulated inflammatory response at high doses [7].

The anticancer potential of silymarin is well known, as detailed in a review by Agarwal et al. [8]. The mode of action, including its antiproliferative effect mediated by influencing the cell cycle, suppression of apoptosis, and inhibition of cell-survival kinases, as well as its anti-inflammatory effect mediated by inhibition of inflammatory transcription factors, is also relatively well studied. The ability of silymarin to suppress angiogenesis and increase the sensitivity of tumors to chemotherapeutic agents and its chemopreventive effect against various promoters of carcinogenesis also contribute to its anticancer effect [8]. Although most studies examine the effect of complex silymarin, in some works, the effects of individual components of the silymarin complex have been compared, especially silybin as the main component and its derivatives, and significant differences in their efficacy were found [9,10]. Other studies investigating silybin’s activity indicate dual effects on the proliferation and immune response of different cell types in a dose-dependent manner [11,12]. However, there is a lack of studies comparing the effect of silybin on the same type of healthy and tumor cells, especially intestinal, partly due to the limited availability of non-transformed cell lines from different tissues. Therefore, our study aimed to investigate the effect of different concentrations of silybin on healthy intestinal porcine epithelial cell line-1 (IPEC-1) and the cell line of the human epithelial colorectal adenocarcinoma (CaCo-2).

## 2. Materials and Methods

### 2.1. Isolation of Silybin A/B

We are grateful for the provision of Silybin A/B by Dr. David Biedermann, Institute of Microbiology of the Czech Academy of Sciences, in the Laboratory of Biotransformations, Prague. The preparation of silybin from silymarin has been described previously [13]. Briefly, 200 mg of silymarin (Liaoning Senrong Pharmaceutical Co., Panjin, China) was suspended in 10 mL of methanol. The insoluble silybin was filtered, and the filtrate was collected and applied to a SEPHADEX HL-20 colony (Sigma Aldrich, Schnelldorf, Germany). The isocratic method with a mobile phase of water/methanol/cyanomethane/formic acid in the ratio 2/37/61/0.1 was used. The flow rate of the mobile phase was 1.2 mL/min, and the detection was performed spectrophotometrically at 285 nm SYNERGY HT (Biotek, Winooski, VT, USA). The purity of the obtained silybin (mixture of two diastereoisomers A and B) was 95 %, as determined by HPLC PDA. For further analyses, silybin was dissolved in dimethyl sulfoxide (Sigma Aldrich, Germany) to a 100 mM stock solution, which was subsequently diluted in the appropriate culture media.

### 2.2. Cell Cultures

The intestinal porcine epithelial cell line-1 (IPEC-1), representing healthy intestinal cells, and the tumor cell line of the human epithelial colorectal adenocarcinoma (CaCo-2) were used as culture models. The IPEC-1 cells were originally isolated from the jejunum of a neonatal, unsuckled piglet and were maintained as a continuous line of non-transformed intestinal cells. They were kindly provided by Dr. Juan José Garrido from the Department of Genetics and Animal Breeding, University of Cordoba, Spain. The CaCo-2 cells (HTB-37^TM^—derived from human colon adenocarcinoma) were purchased from the American Type Culture Collection (ATCC, Manassas, VA, USA). The IPEC-1 cell line was maintained in a medium containing Dulbecco’s Modified Eagle Medium: Nutrient Mixture F-12 (DMEM/F-12; Sigma-Aldrich, St. Louis, MO, USA), supplemented with 5% heat-inactivated fetal bovine serum (FBS; Sigma-Aldrich, USA), 10 µg/mL of insulin, 10 µg/mL of transferrin, 10 ng/mL of selenium (Lonza, Basel, Switzerland), 5 ng/mL of an epidermal growth factor (BD Biosciences, San Jose, CA, USA) and 50 µg/mL of gentamicin (BioSera, Marikina, Philippines). The cells were incubated at 37 °C in a fully humidified atmosphere under 5% CO_2_.

The CaCo-2 cell line was cultured in DMEM/F-12, 10% heat-inactivated fetal bovine serum (Lonza, Switzerland), 1% amphotericin B (BioSera, Philippines), and gentamicin (50 μg/mL; BioSera, Philippines) at 37 °C in a fully humidified atmosphere under 5% CO_2_.

After the formation of a sub-confluent cell monolayer, the passage was performed. Cell viability was greater than 95% before each experiment. In addition, the PCR method was used to regularly check both cell lines for the absence of mycoplasma contamination [14].

### 2.3. Metabolic Viability Assay

The viability of cells measured as the metabolic viability after exposure to silybin was evaluated by MTS (3-(4,5-dimethylthiazol-2-yl)-5- (3-carboxymethoxyphenyl)-2-(4-sulfophenyl)-2H tetrazolium) colorimetric assay (CellTiter 96^®^ Aqueous One Solution Cell Proliferation Assay, Promega, Madison, WI, USA). The IPEC-1 and CaCo-2 cells were separately seeded in standard 96-well microtiter plates (TPP, Trasadingen, Switzerland) at a density of 3 × 10^4^ cells/well in 100 µL of the medium. After 24 h, a monolayer was formed. Subsequently, silybin was added in different concentrations (5, 10, 20, 40, and 80 µM) to 100 µL of medium per well. The untreated control cells contained a silybin-free medium. The cells were incubated at 37 °C in a fully humidified atmosphere under 5% CO_2_ for 4 and 24 h. Then, 20 µL of MTS solution was added to each well, and absorbance was measured at 490 nm using a microplate reader Synergy HT (Biotek, USA). The absorbance of control wells was considered as 100%, and the results were expressed as a percentage of untreated control. The experiment was performed twice, and each sample was run in duplicates.

### 2.4. Cell Cycle Analysis

The effect of silybin on the IPEC-1 and CaCo-2 cell cycle progressions was assessed by flow cytometry. IPEC-1 and CaCo-2 cells were separately seeded at a density of 2.25 × 10^5^ cells/well in 1 mL of medium in standard 24-well microtiter plates (Orange Scientific, Braine-l’Alleud, Belgium). After 24 h, the cell monolayer was treated with silybin at final concentrations of 5, 10, 20, 40, and 80 μM. The untreated control cells contained medium without silybin. After 4 and 24 h of incubation, the cells were harvested following detachment with Accutase solution (ThermoFisher Scientific, Waltham, MA, USA), washed in ice-cold phosphate-buffered saline (PBS; MP Biomedicals, Illkirch-Graffenstanden, France), fixed with ice-cold 60 % ethanol, and stored at 4 °C overnight. The fixed cells were washed in PBS and resuspended in a staining solution consisting of 25 mg/L propidium iodide and 100 mg/L ribonuclease A (Sigma-Aldrich, USA) in PBS. Subsequently, the cells were incubated for 1 h at 37 °C in the dark. Analysis was performed in a flow cytometer BD FACS Canto (BD Biosciences, USA) using BD FACS Diva software (BD Biosciences, USA). After excluding doublets and aggregates from the analysis, the results were evaluated in a histogram (counts versus FL-2-A) as a percentage of cells in the subG1, G0/G1, S, and G2/M phases of the cycle (Figure S1). The experiment was conducted in duplicates in two independent experiments.

### 2.5. Mitochondrial Membrane Potential

Mitochondrial membrane potential (MMP) is closely linked to the intrinsic pathway of apoptosis and was evaluated by the method described previously [15]. Both cell lines were treated with SB as described previously and, immediately after detachment, were washed with PBS and used to measure mitochondrial membrane potential (∆ψm) using Rhodamine 123 fluorescent dye (Sigma-Aldrich, USA), which is taken by mitochondria of living cells. The cells were incubated with Rhodamine 123 (5 µM final) and prepared as a stock solution (1 mg/mL in ethanol) for 20 min at 37 °C. The supernatant was removed after pelleting the cells, and MMP was measured in 150 µL of PBS by flow cytometry with the excitation of Rhodamine 123 at 505 nm and emission at 535 nm. Data were obtained using a FACS Canto flow cytometer (Becton Dickinson Biosciences, USA), analyzed using FACS Diva software, and expressed as the mean fluorescence intensity of Rhodamine 123.

### 2.6. Annexin V/Propidium Iodide Apoptosis Assay

The cell lines were treated in the same way as in the previous assays to examine the concentration-dependent effect of silybin on apoptosis. Apoptosis was detected using a BD Pharmingen Annexin V-FITC Apoptosis Detection Kit (APO Alert Annexin V, ClonTech, CA, USA) according to the manufacturer’s instructions. Briefly, the treated cells were centrifuged for 10 min to remove the medium, then washed and resuspended in 200 µL of the binding buffer. Apoptotic cells were detected after staining with 5 µL of Annexin V and 5 µL of propidium iodide solutions at room temperature in the dark for 15 min. Analysis was performed by flow cytometry using a BD FACS Canto flow cytometer (Becton Dickinson Biosciences), and the data were analyzed using FACS Diva software. The data are expressed as the proportions of live cells, early-stage cells, and late-stage/dead cells and are expressed in % of the total cells (Figure S1).

### 2.7. RNA Isolation and cDNA Synthesis

IPEC-1 and CaCo-2 cells were seeded separately in standard 24-well microtiter plates (Orange Scientific, Belgium) at a 5 × 10^5^ cells/well density in 1 mL of medium. After monolayer forming, the cells were treated as described in Section 2.4. They were incubated at 37 °C in a fully humidified atmosphere under 5% CO_2_ for 4 and 24 h. Total RNA was isolated using a PureZOL^TM^ RNA Isolation Reagent (Bio-Rad, Hercules, CA, USA) according to manufacturing instructions. RNA purity and concentration were determined spectrophotometrically at 260/280 nm by Nanodrop 8000 (Thermo Scientific, USA). Subsequently, the isolated RNA was transcribed to cDNA by the QuantiTect Reverse Transcription Kit (Qiagen, Hilden, Germany). The resulting cDNA was used as a template in qPCR.

### 2.8. Gene Expression Analysis (qPCR)

We determined the gene expression of interleukin-1 (IL-1), IL-6, transforming growth factor beta (TGF-β), and tumor necrosis factor-alpha (TNF-α). GAPDH was used as a reference gene. The sequences of primers used in this experiment are shown in Table 1. The PCR reaction was performed in 10 μL reactions, each consisting of the SYBR green master mix (BioRad, USA), 0.5 μM of both primers, and 40 ng/μL of cDNA. All of the reactions contained negative control without a cDNA template. Quantitative PCR analysis was determined using a BioRad CFX thermocycler (BioRad, USA). The experimental protocol was comprised of denaturation at 95 °C for 5 min and amplification in 40 cycles, which consisted of 4 steps: denaturation at 94 °C for 30 s, hybridization at 60 °C for 30 s and extension at 72 °C for 30 s with measurement of fluorescence. The melt curve was analyzed at 72 °C for 15 min after the final extension. Reaction efficiency was 100 ± 5 for each assay. Relative expression was calculated by the 2^−ΔΔCT^ method. The experiment was conducted in duplicates in two independent experiments.

### 2.9. Statistical Analysis

The results were evaluated using GraphPad Prism 9.0.0 software (GraphPad Software, San Diego, CA, USA) by the Shapiro–Wilk normality test, followed by one-way analysis of variance (ANOVA) and Dunnett’s multiple comparisons test. The results are expressed as mean ± standard deviation (SD) from two independent experiments with duplicates.

## 3. Results

### 3.1. Metabolic Viability Assessment

**Figure 1:**
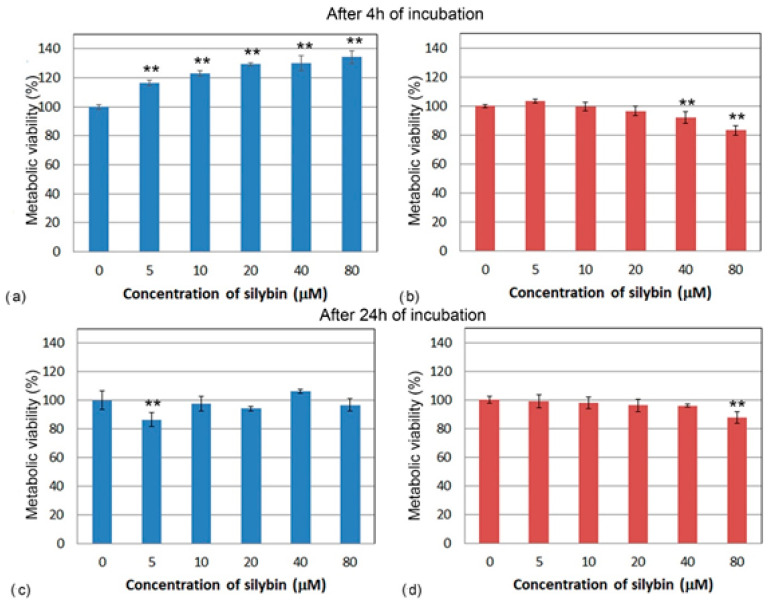
Metabolic viability (%) of (**a**,**c**) IPEC-1 cells (blue bars) and (**b**,**d**) CaCo-2 cells (red bars) treated with different concentrations of silybin (n = 4) for 4 h (**a**,**b**) and 24 h (**c**,**d**). ** significantly different from untreated cells at the level of significance of *p* ˂ 0.01.

To determine the direct effect of silybin on the viability of IPEC-1 and CaCo-2 cells after 4 and 24 h of exposure, we used a colorimetric MTS assay. After 4 h of incubation, the metabolic viability of silybin-treated IPEC-1 cells was significantly higher compared to the control for each tested concentration and elevated with increasing silybin concentration (Figure 1a). However, after 24 h of incubation, except for the 5 µM concentration (Figure 1c), silybin did not significantly influence the cell’s viability. By contrast, after 4 and 24 h, a significant decline in the metabolic viability of CaCo-2 cells compared to the control group was observed at the higher concentrations (40 and 80 µM) (Figure 1b,d).

### 3.2. Analysis of Cell Cycle

**Figure 2:**
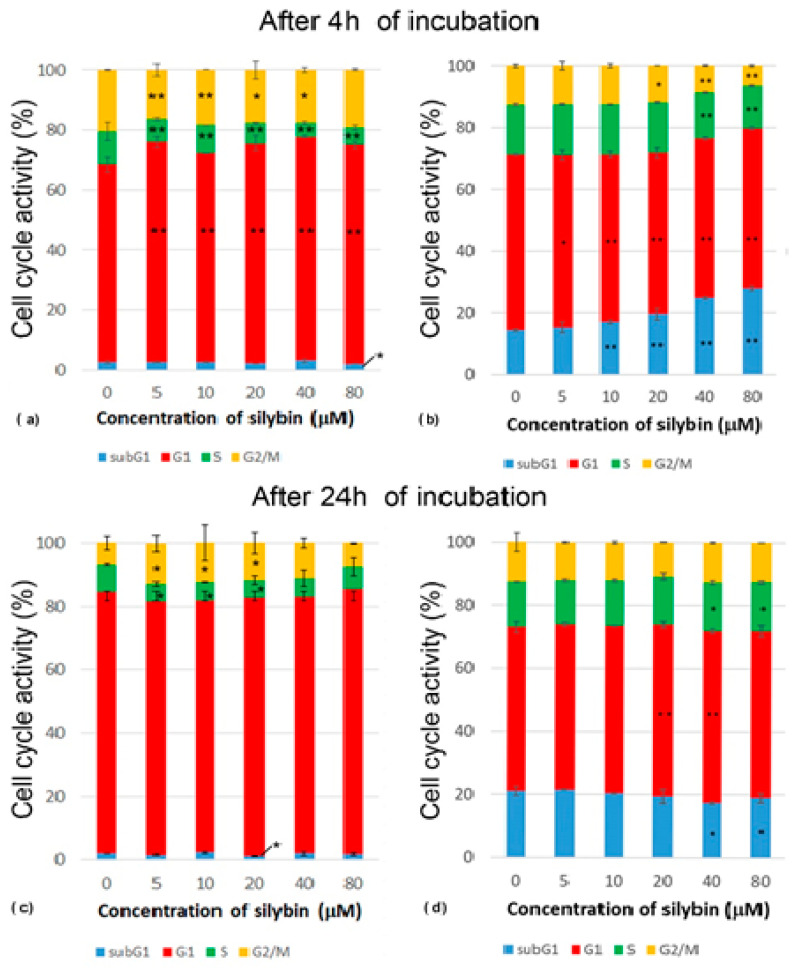
Effect of different concentrations of silybin on the cell cycle phases (%) in IPEC-1 cells after (**a**) 4 h and (**b**) 24 h incubation and in CaCo-2 cells (**c**) after 4 h and (**d**) 24 h incubation. The stars indicate significant difference from untreated cells; the level of significance: * *p* ˂ 0.05 and ** *p* ˂ 0.01.

After 4 h of incubation with silybin, there was a significantly increased percentage of IPEC-1 cells in their G1 phase, while a reduced percentage of cells in the S phase of the cell cycle was measured in all experimental groups compared to the control. Groups treated with concentrations of silybin ranging from 5 to 40 μM had a lower number of cells in the G2/M phase when compared to the control (Figure 2a). After 24 h of incubation, we found a decreased percentage of cells in the S phase at concentrations of 5–20 µM and an increased percentage of cells in the G2/M phase at concentrations of 5–40 µM in comparison with the control group. This was caused by reduced mitosis after 4 h of incubation and the subsequent creation of a monolayer (Figure 2c). The CaCo-2 cell line showed a concentration-dependent increase in cells in the subG1 phase. The proportions of cells in the G1 phase were significantly lower in all of the experimental groups compared to the control. The highest concentrations of silybin (40 μM and 80 μM) led to a decrease in the number of cells in the S phase, the inhibition of mitosis (G2/M phase), and the biggest shift towards the subG1 phase (Figure 2b). After 24 h incubation of silybin with CaCo-2 cells with silybin, we noted a significantly decreased percentage of cells in the subG1 phase at concentrations of 40 and 80 µM and an increased proportion of cells in the G1 phase at concentrations of 20 and 40 µM in comparison to the control group (Figure 2d). These data indicate that silybin interfered with some regulatory factors in cancer CaCo-2 cells but not normal IPEC-1 cells involved in the mitotic process.

### 3.3. The Effect of Silybin on the Induction of Apoptosis

**Figure 3:**
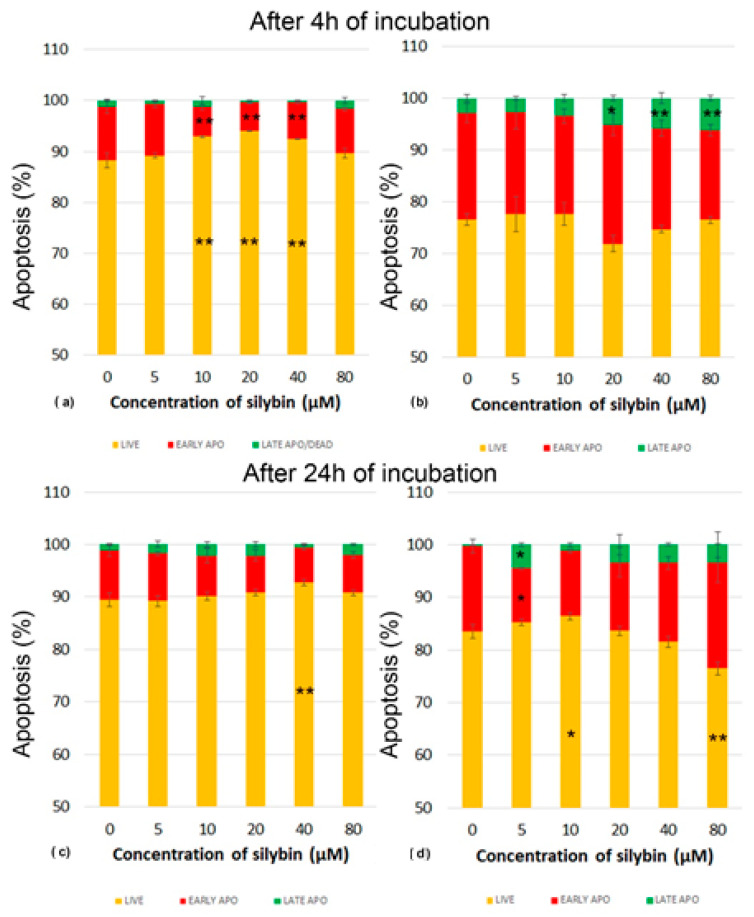
Effect of different concentrations of silybin on the apoptosis in IPEC-1 cells after (**a**) 4 h and (**b**) 24 h incubation and in CaCo-2 cells (**c**) after 4 h and (**d**) 24 h incubation. The stars indicate significant difference from untreated cells; the level of significance: * *p* ˂ 0.05 and ** *p* ˂ 0.01. The orange parts of the bars represent living cells, the red parts represent cells in the early stage of apoptosis, and the green parts represent cells in late apoptosis or dead cells.

Next, we analyzed whether various concentrations of silybin can influence the process of programmed cell death-apoptosis in IPEC-1 and CaCo-2 cells. Apoptosis was monitored by flow cytometry. After 4 h of incubation, silybin seemed to exert a cytoprotective effect between concentrations of 10 and 40 µM on IPEC-1 cells, recorded as significantly higher numbers of live cells (Figure 3a). After 24 h of incubation, this effect was seen only at the concentration of 40 µM. The proportions of IPEC-1 cells in the early stage of apoptosis were minimally influenced (Figure 3c).

In contrast, CaCo-2 cells were highly sensitive to the applied in vitro cultivation system considered the standard. In comparison with IPEC-1 cells, the higher CaCo-2 cell numbers entered the early stage of apoptosis after 4 h of incubation. The cultivation of cells with silybin at concentrations between 20 and 80 µM resulted in significantly higher proportions of late apoptosis/dead cells and a decrease in live cells (Figure 3b). After 24 h cultivation, we recorded elevated numbers of live CaCo-2 cells and early-stage apoptotic cells compared to those numbers after 4 h in the control samples. Silybin at the 5 µM concentration induced a significantly higher proportion of dead cells and, at the same time, a lower percentage of cells in early apoptosis. A similar trend was noted for silybin concentrations of 10–40 µM, but without statistical significance at the 80 µM concentration of silybin, a higher percentage of late apoptotic/dead cells was accompanied by a significantly lower number of live cells (Figure 3d).

### 3.4. Mitochondrial Membrane Potential

**Figure 4:**
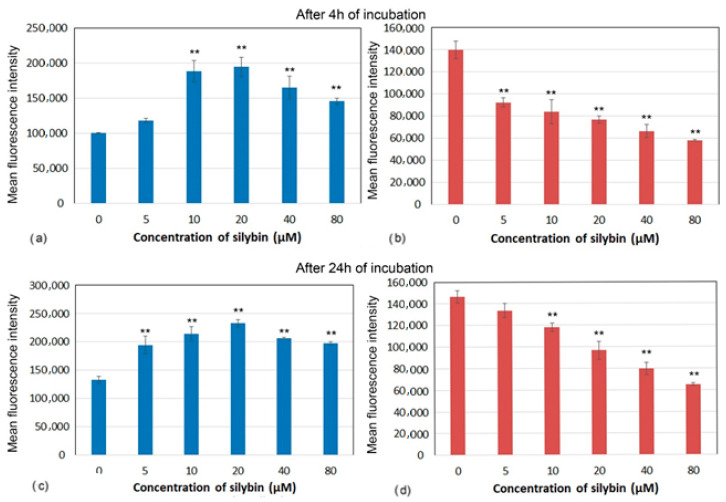
Membrane potential (mean fluorescence intensity) of (**a**,**c**) IPEC-1 and (**b**,**d**) CaCo-2 cells treated with different concentrations of silybin (n = 4) after 4 h (**a**,**b**) and 24 h incubation (**c**,**d**). The stars indicate significant difference from untreated cells; the level of significance: ** *p* ˂ 0.01.

Decreases in mitochondrial membrane potential (MMP) have been associated with mitochondrial dysfunction that could lead to cell death. Marked differences in MMP after incubation with silybin on CaCo-2 cells and IPEC-1 cells were recorded. After 4 and 24 h of incubation, silybin significantly increased MMP in IPEC-1 cells with the highest elevation between 10 and 20 µM (Figure 4a,c). In contrast, MMP in CaCo-2 cells showed opposite kinetic after incubation with silybin. In comparison with control cells, a significant concentration-dependent decrease in MMP was recorded from 5 µM after 4 h and from 10 µM after 24 h of incubation (Figure 4b,d).

### 3.5. The Effect of Silybin on the Gene Expression of Selected Cytokines

**Figure 5:**
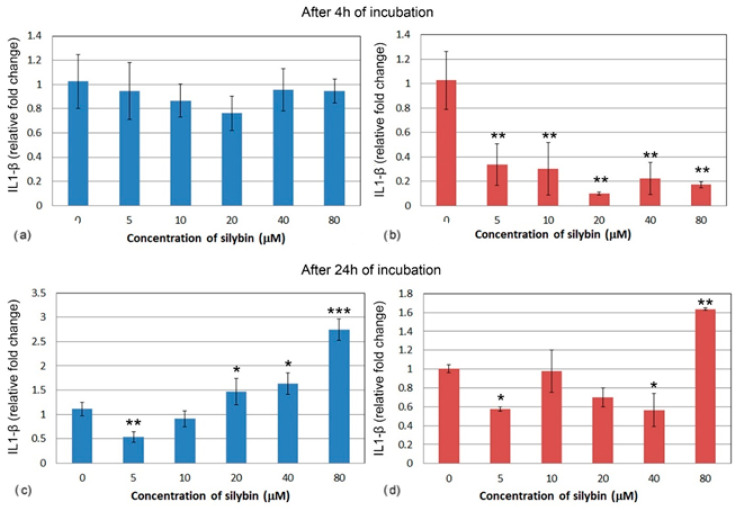
Relative gene expression of IL-1 (ΔΔCt) in (**a**,**c**) IPEC-1 cells (blue bars) and (**b**,**d**) CaCo-2 cells (red bars) treated with different concentrations of silybin (n = 4) after 4 h (**a**,**b**) and 24 h incubation (**c**,**d**). The stars indicate significant difference from untreated cells; the level of significance: * *p* ˂ 0.05, ** *p* ˂ 0.01, *** *p* ˂ 0.001.

The relative gene expression for interleukin 1 (IL1) did not change significantly in IPEC-1 cells after 4 h cultivation with silybin (Figure 5a). In CaCo-2 cells, a significant decrease in IL1 gene expression was noted in all of the experimental groups when compared to the control group. A stronger effect was observed at the concentrations of 20, 40, and 80 μM. (Figure 5b). After 24 h of cultivation, a relative gene expression of IL1 was significantly increased in IPEC-1 cells that were treated with 20, 40, and 80 μM of silybin when compared to the control, with the greatest change in the group that was treated with the highest concentration (Figure 5c). In all of the CaCo-2 cells, except for the group that received the highest concentration of silybin (80 μM) (Figure 5d), the overall relative gene expression of IL1 was decreased.

**Figure 6:**
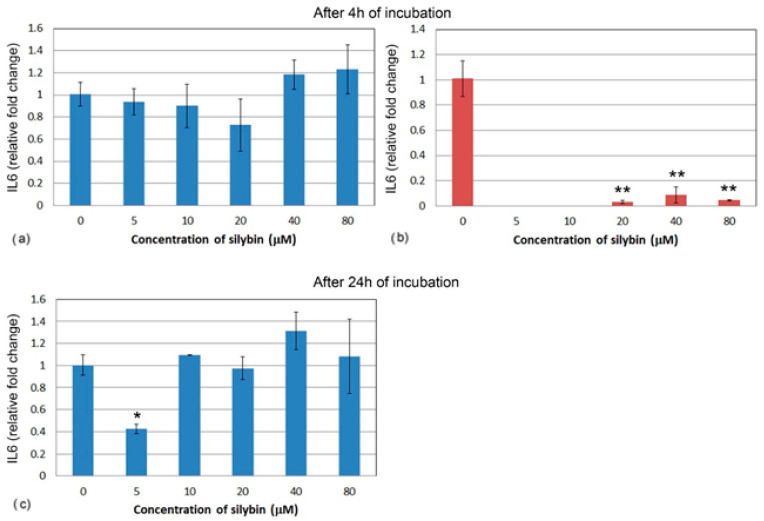
Relative gene expression of IL6 (ΔΔCt) in (**a**,**c**) IPEC-1 (blue bars) and (**b**) CaCo-2 cells (red bars) treated with different concentrations of silybin (n = 4) after 4 h cultivation (**a**,**b**) and 24 h (**c**). The stars indicate significant difference from untreated cells; the level of significance: * *p* ˂ 0.05, ** *p* ˂ 0.01.

Similarly, the relative gene expression of IL6 did not significantly change in IPEC-1 cells after 4 h (Figure 6a). At the lower concentrations of silybin, IL6 gene expression was not measurable in CaCo-2 cells, and at the higher concentrations, relative gene expression was significantly lowered in comparison with the control (Figure 6b). After 24 h, IL6 gene expression was significantly lower in the group of IPEC-1 cells that were treated with 5 μM of silybin. No significant changes were observed in the other experimental groups (Figure 6c). The relative gene expression of IL6 was not measurable in CaCo-2 cells; therefore, it is not presented in Figure 6.

**Figure 7:**
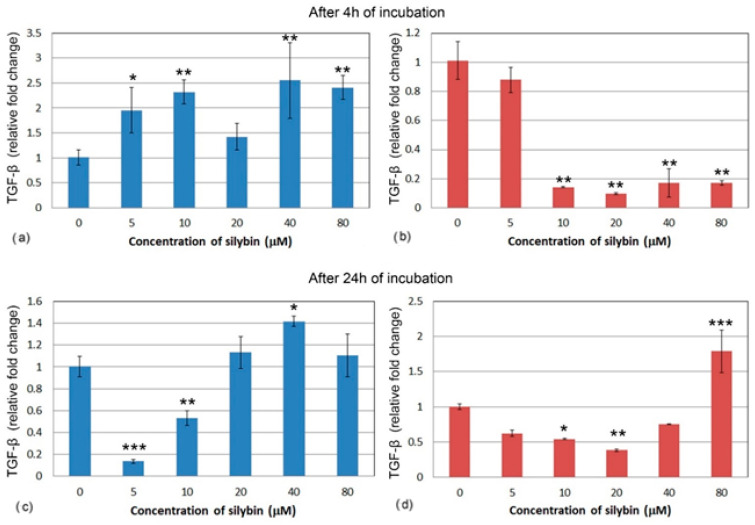
Relative gene expression of TGF-β (ΔΔCt) in (**a**,**c**) IPEC-1 cells (blue bars) and (**b**,**d**) CaCo-2 cells (red bars) treated with different concentrations of silybin (n = 4) after 4 h (**a**,**b**) and 24 h (**c**,**d**) cultivation. The stars indicate significant difference from untreated cells; the level of significance: * *p* ˂ 0.05, ** *p* ˂ 0.01, *** *p* ˂ 0.001.

After 4 h, all of the IPEC-1 cells, with the exception of the 20 μM group (Figure 7a), showed a significant increase in relative gene expression for TGF-β after silybin treatment. Gene expression of TGF-β was different in the CaCo-2 cells. All of the concentrations except for the 5 μM concentration caused a significant decrease in TGF-β relative gene expression (Figure 7b). In IPEC-1 cells that were treated with silybin for 24 h, a significant decrease in gene expression for TGF-β was recorded in groups that received silybin at 5 and 10 μM concentrations. The other groups showed a slight increase in gene expression (Figure 7c). Except for the 80 μM group, an overall decrease in CaCo-2 cells was observed after 24 h. In the group that received the highest dose, a significant increase was measured. At the concentration of 20 μM, silybin caused the most significant decrease in gene expression (Figure 7d).

**Figure 8:**
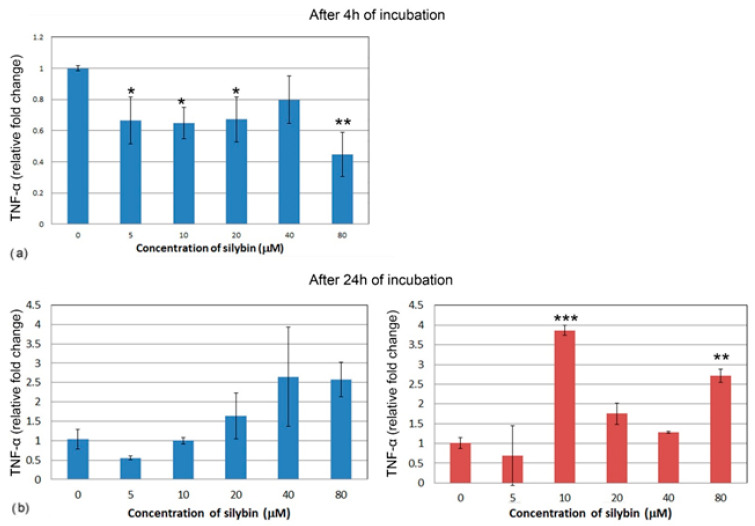
Relative gene expression of TNF-α (ΔΔCt) in the (**a**,**b**) IPEC-1 cells (blue bars) and (c) CaCo-2 cells (red bars) treated with different concentrations of silybin (n = 4) after 4 h (**a**) and 24 h cultivation (**b**,**c**). The stars indicate significant difference from untreated cells; the level of significance: * *p* ˂ 0.05, ** *p* ˂ 0.01, *** *p* ˂ 0.001.

After 4 h of incubation, the IPEC-1 cells showed a decrease in relative expression for TNF-α in all of the concentrations of silybin except for the 40 μM concentration, with the most significant change in the 80 μM group (Figure 8a). CaCo-2 cells did not show TNF-α gene expression after 4 h of incubation; therefore, it is not presented here. Following 24 h of incubation, no significant change was recorded in the IPEC-1 cells, although an overall increase was noted (Figure 8b). In CaCo-2 cells, a significant increase in gene expression of TNF-α was found in the groups treated with 10 μM and 80 μM of silybin (Figure 8c).

## 4. Discussion

We studied the effect of silybin, the main component of the silymarin complex, on metabolic viability, cell cycle, apoptosis, mitochondrial membrane potential, and gene expression of selected cytokines in healthy IPEC-1 cells and cancer cell line CaCo-2. Given the fact that there is limited knowledge about the effects of individual components of the silymarin complex on intestinal cells, this study provides an in vitro baseline result for further in vivo experiments.

After 4 h of incubation, silybin had a stimulating effect on the metabolic viability of healthy porcine cells, even at high concentrations—40 and 80 μM. A similar effect was also observed in other studies where silibinin was complexed with metals such as copper. The complex that was produced stimulated the differentiation of osteoblast and angiogenesis in vitro in healthy cells [16].

On the other hand, silybin significantly suppressed the metabolic viability of intestinal cancer cells. These results indicate that silybin has a positive effect on healthy cells and a suppressive effect on cancer cells. After 24 h of incubation, the effect on both types of cell lines was not significant because a monolayer of cells had already been created. Higher concentrations of silybin are more effective at destroying cancer cells, as is evident from an analysis of the cell cycle. Concentrations of 40 and 80 μM caused a significant shift towards cells in the subG1 phase, which represents the population of damaged and apoptotic cells. After 24 h incubation, we noticed a reduced effect of silybin on the cell cycle of cancer CaCo-2 cells in comparison to 4 h incubation, while the percentage of cells was reduced in S and G2/M phases. This result is in line with other studies using flavonoids, although in studies that used flavonoid metal complexes to study antitumor activities, the concentration of used molecules was lower than in our experiment with a strong suppressing effect on cancerous cell lines, as reviewed by Khater et al. [17].

Deep et al. [18] studied the effect of individual components of the silymarin complex on the cell cycle of human prostate cancer cells and used concentrations of 60 and 90 μM of silybin A, silybin B, isosilybin A, isosilybin B, silydianin, silychristin, and isosilychristin. The results showed that 48 h incubation with silybin B caused a significant increase in apoptosis of cancerous cells. When they measured mitotic activity, they noted that after 72 h of incubation with silybin A and B, the number of cells in the G1 phase was increased but reduced in the S phase when compared to the control. Our results showed the same effect of higher concentrations of silybin at 40 and 80 μM. Deep et al. [18] further measured the expression of molecules called cyclins, which are important in the regulation of the cell cycle. A significantly decreased expression of cyclins D1, D3, E, A, and B1 in cancerous cells was recorded after incubation of cells with 90 μM silybin A and B. The authors then compared individual substituents of the silymarin complex and found that only silybin A and B and isosilybin A and B had a down-regulating effect on the cell cycle, while silydianin, silychristin, and isosilychristin did not affect the cell cycle of cancer cells. The important aspect that affects the final effect of selected components is their chemical structure. Although these compounds have an identical formula (C_25_H_22_O_10_) and a molecular weight of 482.1, their different stereochemical structure and condensation products probably had a different effects on the mitotic activity of cancerous cells. Salucci et al. [19] studied the effect of selected flavonoids (epicatechin, epigallocatechin gallate, gallic acid, and quercetin-3-glucoside) on the cell cycle of the CaCo-2 cell line as flavonoids have been shown to exert a potentially protective effect against colon carcinoma. The results revealed that only epigallocatechin gallate and gallic acid (100 μM) inhibited the cell cycle of Caco-2 cells. The authors, therefore, assume that galloyl moiety is required for their antiproliferative effect. It confirms that the chemical structure is a critical factor in the ability of flavonoids to regulate mitotic activity.

Mitochondrial membrane potential (MMP, ∆ψm) is generated by the electric potential across the inner mitochondrial membrane, and measuring MMP is useful for evaluating mitochondrial function. A decrease in MMP can be induced by xenobiotics that dissipate it directly (uncouple) or by xenobiotics that disrupt other cellular processes or affect different mitochondrial functions, including respiration, tricarboxylic acid (TCA) cycle, fatty acid β oxidation, and pyruvate or fatty acid uptake. A decrease in MMP may also be linked to apoptosis pathways [20]. In our study, the proportions of live IPEC-1 cells were higher than CaCo-2 cells after 4 and 24 h of cultivation in the control group, indicating better adaptability of normal intestinal cells to ex vivo cultivation conditions. Silybin retained the viability of IPEC-1 cells and elevated numbers of live cells on account of early apoptotic cells and dead cells. However, CaCo-2 cells were more sensitive to silybin, and the higher concentrations caused a shift in the early stage and late stage of apoptosis in elevated numbers of cancer cells. The intracellular signaling pathways leading to the onset of apoptosis are almost as diverse as the number of compounds capable of inducing this unique form of cell death. Surprisingly, almost all of the mechanisms characterized so far appear to involve signaling via the mitochondria [21]. In our study, the higher numbers of live IPEC-1 cells in silybin-treated groups correlated with significant MMP elevation after 4 and 24 h of incubation. These findings suggest the direct stimulating effect of silybin on mitochondrial membrane integrity, probably due to the free radical scavenging activity of this flavonolignan. Interestingly, silybin significantly decreased MMP in CaCo-2 cells in correlation with the higher proportions of dead cells and cells in early apoptosis. In general, cancer cells gain the energy for growth via different metabolic pathways than normal cells, mostly via glycolysis, and prefer hypoxic conditions [22]. These physiological differences between normal intestinal cells and cancer cells probably accounted for the deleterious effect of silybin on mitochondrial membrane integrity and function in cancer cells and, consequently, on apoptosis. Esselun et al. [23] showed that silybin A significantly weakened nitrosative stress in neuronal PC12 and HepG2 liver cells. Low concentrations maintained not only protective properties but also increased basal mitochondrial membrane potential (MMP) and adenosine triphosphate (ATP) levels.

IL1 is a well-known pro-inflammatory cytokine in mammals. Kang et al. [24] demonstrated that silymarin suppresses the synthesis of IL1 in macrophages after their stimulation with LPS. Silymarin also has anti-inflammatory, immunomodulatory, and antioxidative effects, as reviewed by Katiyar [25]. Our results showed no changes in the relative gene expression of IL1 in healthy intestinal cells after 4 h of incubation with silybin. However, relative gene expression of the same interleukin was decreased in the CaCo-2 line. Inflammation is a typical accompanying symptom of carcinogenesis. This also corresponds to previous research on silymarin and its modulating effect on IL1 [26,27], although the reasons behind the decrease in CaCo-2 cells only are unclear. In comparison, after the prolonged cultivation of these cell lines for 24 h, we observed an increase in gene expression for IL1 in healthy cells, but only in groups that received a higher dose of silybin. The level of IL1 in CaCo-2 cells remained decreased even after 24 h, but slightly higher than after 4 h. The highest dose had the same stimulating effect on the synthesis of IL1 as in IPEC-1 cells, proving that it is important to use the correct concentration for health benefits.

Silybin caused a decrease in the relative gene expression of IL6 in the CaCo-2 cell line after 4 h. IL6 is a pro-inflammatory cytokine, and it has been demonstrated that silymarin can decrease its production by the inhibition of the IL-6/STAT3 signaling pathway [28,29]. Other flavonoids (morin, naringenin, and quercetin) also reduced IL6 expression in cells that were stressed by culturing in a high-glucose medium [30]. As with IL1, we did not detect a change in the IPEC-1 cells regardless of silybin concentrations after 4 h. This result is consistent with research in the field of plant extracts, where no change in expression for IL6 was measured after treatment of different types of cell cultures with various flavonoids. However, after the challenge with LPS or mycotoxins, flavonoids had an anti-inflammatory effect and lowered IL6 expression in healthy IPEC-1 cells [31,32,33]. Testing whether silybin has similar properties after the challenge of cells could be the subject of further research; at present, there have been no publications on this topic. After 24 h, we could not measure any expression for IL6 in CaCo-2 cells. In IPEC-1 cells, the only group that received the lowest concentration of silybin (5 μM) showed a significant decrease, while other experimental groups remained unchanged when compared to 4 h cultivation.

Relative gene expression for anti-inflammatory cytokine TGF-β was increased in the turbot gut when fish received a silymarin-enriched diet [34]. We observed a similar increase in gene expression in the IPEC-1 cell line, which represents healthy intestinal tissue, and this increase was concentration-dependent. Interestingly, it was observed that silymarin has a suppressive effect on TGF-β in hepatic cells [35], renal cells [36], and cardiac cells [37] that have been previously damaged. Silybin inhibited TGF-β production in hepatic stellate cells even more strongly than the silymarin complex [38]. In another study, silymarin had a protective effect on renal cells previously stimulated by TGF-β, which is one of the key factors for the induction of interstitial fibrosis and apoptosis. Silymarin reduced the number of apoptotic cells by directly affecting the TGF pathway; in addition, its antifibrotic and antioxidant effects have been confirmed [39]. Similarly, in our experiment, silybin significantly reduced TGF-β gene expression in CaCo-2 cells, even in a concentration of 10 μM. Longer exposure to silybin in low concentrations had a suppressing effect on the synthesis of TGF in IPEC-1 cells, a result that is more in line with previous research. After 24 h, CaCo-2 cells still showed decreased expression in experimental groups with the exception of the 80 μM group, in which it was increased, similar to the previously mentioned IL1.

Wang et al. [40] discovered that silymarin also inhibits the production of inflammatory mediators such as TNF-α and nitric oxide. Our results show a similar effect of silybin on IPEC-1 cells after 4 h, where we observed a decrease in TNF-α gene expression in all of the experimental groups. Although after 24 h, only an insignificant decrease was measured, a slight increase was observed in groups with higher concentrations. Interestingly, the gene expression for TNF-α in CaCo-2 cells after 4 h was below the expression level of the reference gene and increased significantly after 24 h in the two groups. This could be due to the fact that the silymarin complex contains other compounds aside from silybin that have this anti-inflammatory effect.

## 5. Conclusions

In healthy IPEC-1 cells, silybin increases metabolic viability as well as proliferative activities, protects mitochondrial membrane integrity, and thus exerts a cytoprotective effect; it has only a minimal effect on the gene expression of pro-inflammatory cytokines but significantly increases anti-inflammatory TGF-β. In contrast, it has an antiproliferative effect on tumor intestinal CaCo-2 cells and decreases their metabolic viability and mitochondrial membrane potential, which is accompanied by increased apoptosis while significantly reducing the expression of genes for pro-inflammatory interleukins as well as TGF-β. The antiproliferative, cytotoxic, and anti-inflammatory effect of silybin on tumor intestinal cells without a negative effect on healthy cells is a prerequisite for its potential use in the adjuvant therapy of colon cancer.

## Figures and Tables

**Table 1:** The primer sequences used for the qPCR.

**IPEC-1**	**Forward**	**Reverse**
**GAPDH**	CCTGCTTCACCACCTTCTTGA	CCCCAACGTGTCGGTTGT
**IL1 β**	TGGATGGGCGGCTGATTTGAAG	CGAACCCGTGTTGCTGAAGGAG
**IL6**	CAGGGTCTGGATCAGTGCTT	AGCAAGGAGGTACTGGCAGA
**TNF-α**	TAGACCTGCCCAGATTCAGC	AACCTCCTCTCTGCCATCAA
**TGF-β**	ACAACTCCGGTGACATCAAAGG	ACGTGGAGCTATACCAGAAATACAG
**CaCo-2**	**Forward**	**Reverse**
**GAPDH**	GACAAGCTTCCCGTTCTCAG	TCACCAGGGCTGCTTTTAAC
**IL1 β**	TCTTTCAACACGCAGGACAG	TCCAGGGACAGGATATGGAG
**IL6**	AGTGCCTCTTTGCTGCTTTC	TACCCCCAGGAGAAGATTCC
**TNF-α**	GCCAGAGGGCTGATTAGAGA	TCAGCCTCTTCTCCTTCCTG
**TGF-β**	GCGGAAGTCAATGTACAGCTGCCGC	TGAACCGGCCTTTCCTGCTTCTCATG

## Data Availability

The data presented in this study are available on request from the corresponding author.

## Supplementary Materials

The following supporting information can be downloaded at: https://www.mdpi.com/article/10.3390/life13020492/s1, Figure S1: Gating strategy for cell cycle analysis. After gating of the mail cell populations for IPEC-1 and Caco-2 cells and exclusion of doublets and aggregates, cell cycle was analysed based on the position of the individual phases (subG1, G1, S, G2/M) of the cell cycle on the histogram. Figure S2: Gating strategy for apoptosis. After gating of the mail cell populations on dot plots FSC-A versus SSC-A for IPEC-1 and Caco-2 cells and exclusion of doublets and aggregates on dot plot PE-A versus PE-H as showed in Figure S1, apoptosis was analysed based on the position of live, early apoptic, late apoptic/dead cells on dot plot FITC-A (Annexin-V labeled with FITC) versus PE-A (propidium iodide). Gating for apoptosis differs between Caco and IPEC cells due to the high intrinsic autofluorescence of IPEC cells.
