# Supplementary material for: Silybin Showed Higher Cytotoxic, Antiproliferative, and Anti-Inflammatory Activities in the CaCo Cancer Cell Line while Retaining Viability and Proliferation in Normal Intestinal IPEC-1 Cells
Source: Life (Basel). 2023 Feb 10;13(2):492. doi: 10.3390/life13020492 (PMC9964225; doi:10.3390/life13020492)

**Supplementary files S1 and S2**

**Figure S1: Gating strategy for cell cycle analysis.** After gating of the mail cell populations for IPEC-1 and Caco-2 cells and exclusion of doublets and aggregates, cell cycle was analysed based on the position of the individual phases (subG1, G1, S, G2/M) of the cell cycle on the histogram.

A) gating of the mail population of IPEC-1 cells

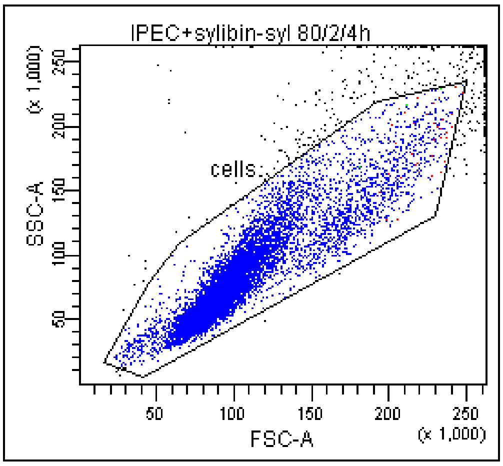


B) gating on singlet IPEC cells

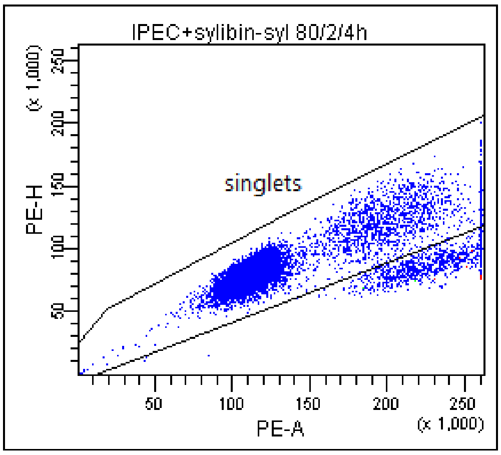


C) IPEC control cells after 4 h incubation

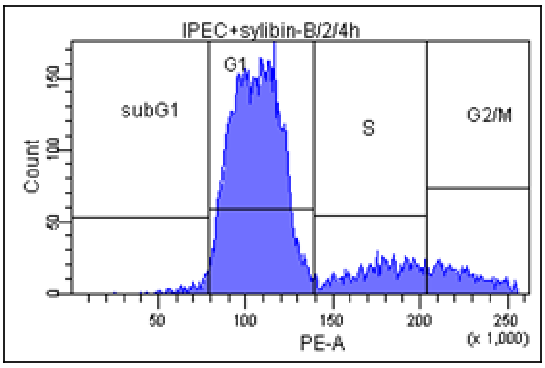


D) IPEC cells treated with 80 uM Silybin after 4 h

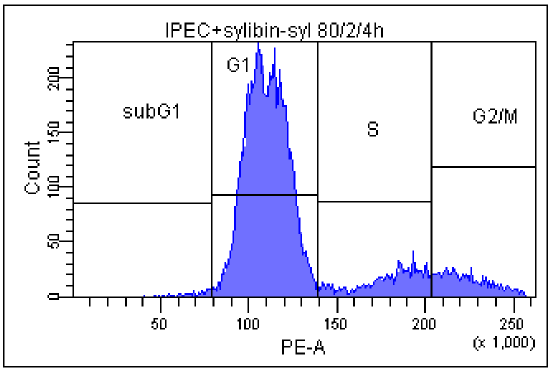


E) IPEC control cells after 24 h incubation

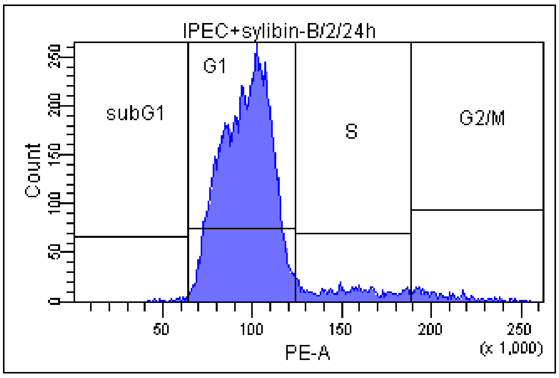


F) IPEC cells treated with 80 uM Silybin after 24 h

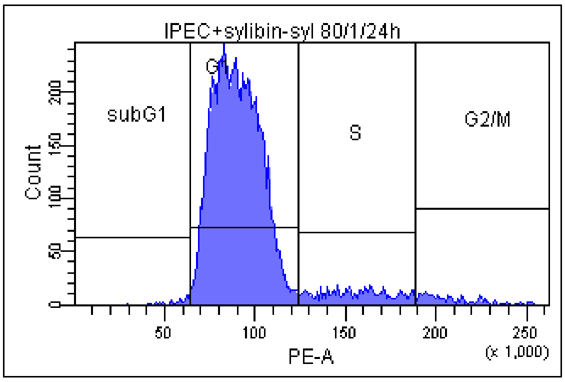

G) gating of the mail population of Caco-2 cells

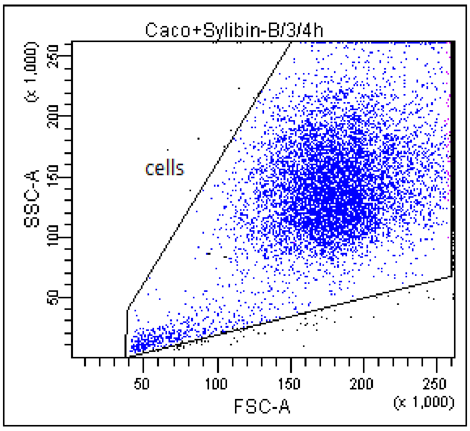


H) gating on singlet Caco cells

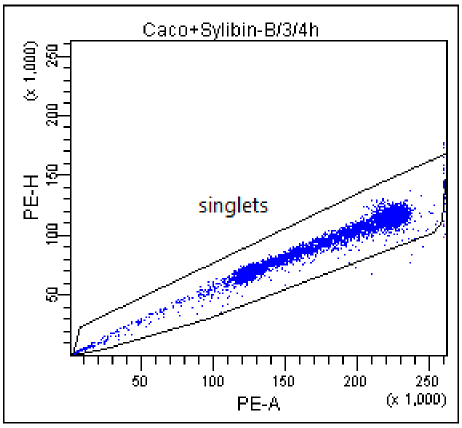


I) Caco control cells after 4 h incubation

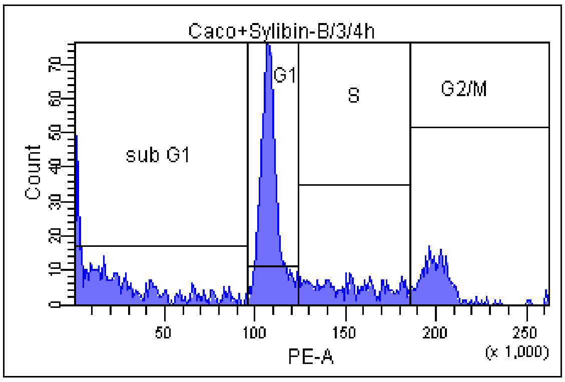


J) Caco cells treated with 80 uM Silybin after 4 h

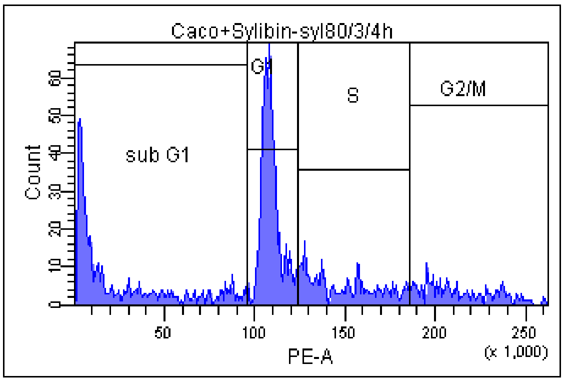


K) Caco control cells after 24 h incubation

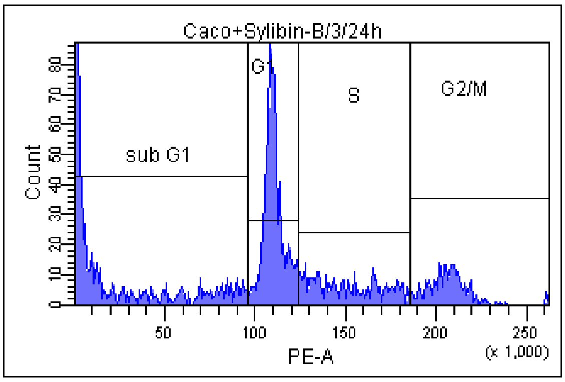


L) Caco cells treated with 80 uM Silybin after 24 h

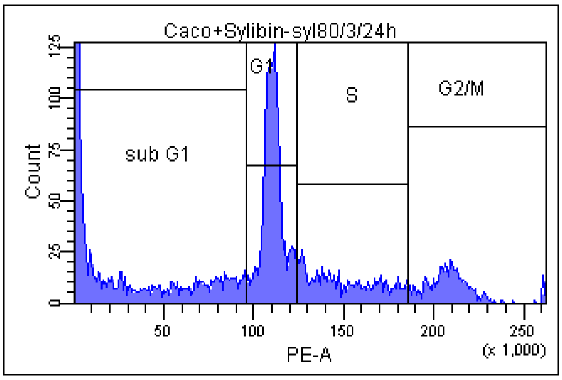

**Supplementary file S2**

**Figure S2: Gating strategy for apoptosis.** After gating of the mail cell populations on dot plots FSC-A versus SSC-A for IPEC-1 and Caco-2 cells and exclusion of doublets and aggregates on dot plot PE-A versus PE-H as showed in Figure S1, apoptosis was analysed based on the position of live, early apoptic, late apoptic/dead cells on dot plot FITC-A (Annexin-V labeled with FITC) versus PE-A (propidium iodide). Gating for apoptosis differs between Caco and IPEC cells due to the high intrinsic autofluorescence of IPEC cells.

A) unlabeled IPEC cells after 4 h incubation

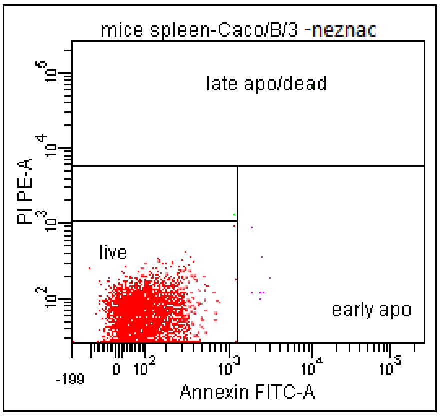


B) Caco control cells after 4 h incubation

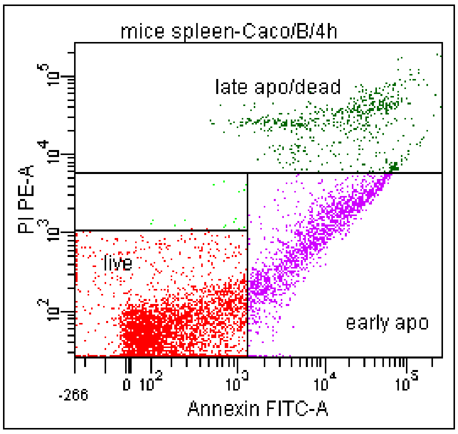


C) Caco cells treated with 80 uM Silybin after 4 h

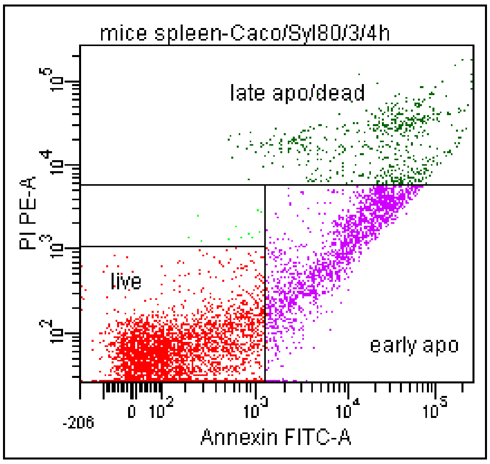


D) Caco control cells after 24 h incubation

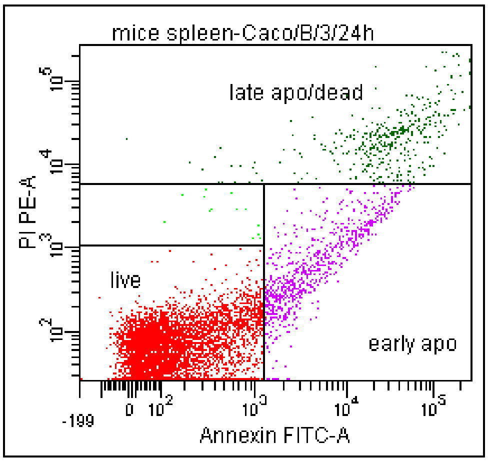

E) Caco cells treated with 80 uM Silybin after 24 h incubation

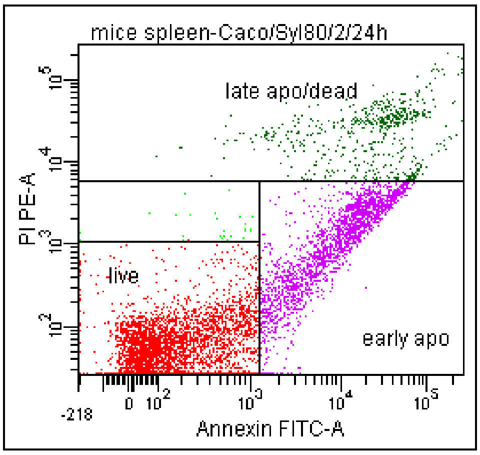


F) Unlabeled unlabeled IPEC control cells with high autofluorescence to FITC and PE (after 4 h incubation)

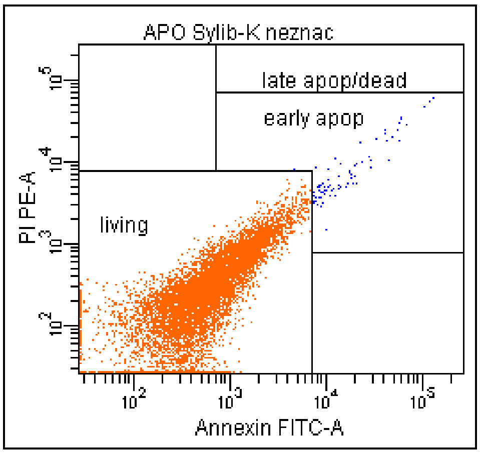


G) IPEC control cells after 4 h incubation

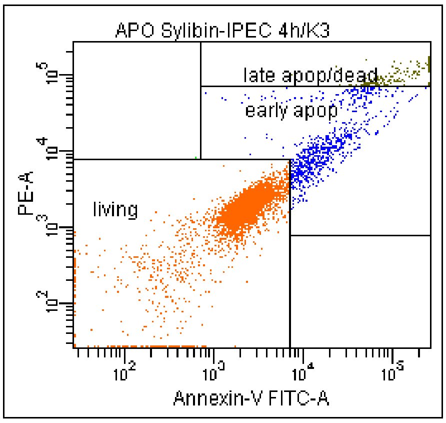


H) IPEC cells treated with 80 uM Silybin after 4 h

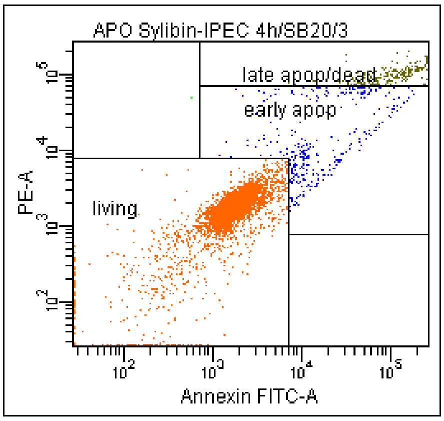

I) IPEC control cells after 24 h incubation

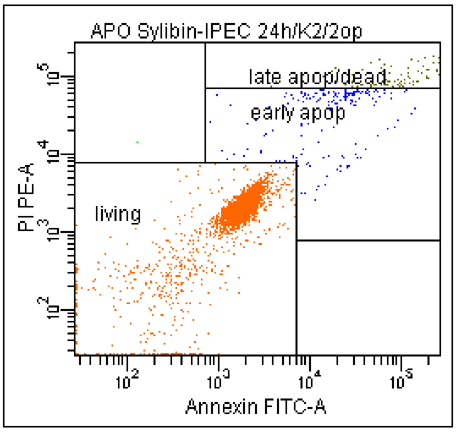


J) IPEC cells treated with 80 uM Silybin after 24 h

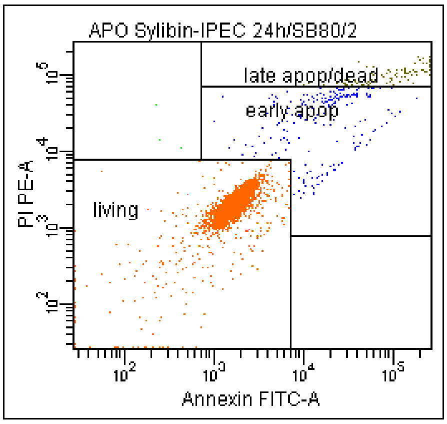

Supplement: Supplementary file 1 [file life-13-00492-s001.zip › life-2092800-supplementary.pdf]
